# The Burden of Dysphagia on Family Caregivers of the Elderly: A Systematic Review

**DOI:** 10.3390/geriatrics3020030

**Published:** 2018-06-10

**Authors:** Ashwini M. Namasivayam-MacDonald, Samantha E. Shune

**Affiliations:** 1 Communication Sciences and Disorders, Adelphi University, Garden City, New York, NY 11530, USA; 2 Communication Disorders and Sciences, University of Oregon, Eugene, OR 97403, USA; sshune@uoregon.edu

**Keywords:** dysphagia, deglutition, caregiver burden, geriatrics, elderly, systematic review

## Abstract

With the rapid increase in the elderly population, there is a simultaneous increased need for care provided by family caregivers. Research in the field of head and neck cancer has indicated that caring for patients with dysphagia can impact a caregiver’s quality of life. Given that many older adults present with dysphagia, one can assume that their caregivers are equally, if not more greatly, affected. The purpose of this systematic review was to examine all relevant literature regarding the caregiver burden in caregivers of community-dwelling older adults with dysphagia. A review of relevant studies published through April 2018 was conducted using search terms related to dysphagia, caregiver burden, and older adults. The search yielded 2331 unique abstracts. Of the 176 abstracts that underwent full review, four were accepted. All reported an increase in caregiver burden due to presence of dysphagia in care recipients. Worsening feeding-related behaviors were associated with burden, and the use of feeding tubes was more frequently associated with “heavy burden”. The presence of dysphagia in community-dwelling older adults is a factor leading to an increased burden among caregivers. Although aspects of dysphagia play a role in the caregiver burden, the specific reasons for the increased burden are unknown. Clinicians should be aware of dysphagia as a source of the burden, and future studies should further define the relationship between dysphagia and the caregiver burden in order to develop comprehensive approaches to care.

## 1. Introduction

Older adults are more susceptible to developing dysphagia (swallowing difficulties) [1,2], which is a contributor to the high physical, psychosocial, and financial costs associated with disease [3]. Dysphagia can result in decreased eating, leading to malnutrition and increased pulmonary compromise and mortality [4,5,6,7]. It can also result in decreased social participation, and increased anxiety and depression, which have been linked to decreased quality of life and increased mortality [8,9,10]. Ultimately, dysphagia does not exist within an isolated individual and may yield psychosocial strain on the entire family unit [11]. Mealtimes and their related rituals foster interpersonal involvement and social connections. Thus, disruptions in daily life stemming from dysphagia likely also affect the psychosocial well-being of an entire family, particularly the primary caregivers [12,13,14,15,16]. 

Caregivers provide valuable support [17], but the influence of dysphagia on caregivers may extend beyond merely support provision. From increased time spent thinking about and preparing two separate meals to limiting shared mealtimes, dysphagia may negatively impact caregivers. These caregivers experience increased feelings of burden, distress, frustration, sadness, isolation, and decreased quality of life [12,13,14,15,16]. Such profound “third-party disability”, or disability of family members stemming from the health condition of their significant other [17,18], suggests the necessity of considering family members both as a piece of a patient’s support system and as individuals with their own needs.

The negative consequences of dysphagia may be further magnified for family caregivers of older adults. Older adults often present with complex health needs and experience increasing limitations in their ability to perform activities of daily living [19]. Caregivers frequently assist these older individuals with a range of healthcare-related activities—up to 28 h per week [20]. Despite this unpaid care saving the American healthcare system approximately $350 billion annually [21], little clinical attention is paid to caregivers. Caregivers that provide substantial healthcare-related help for older adults are significantly more likely to experience emotional, physical, and financial difficulties [20], and experience work productivity loss and reduced participation in valued activities. Family caregivers providing substantial help are also more likely to live with the care recipient (CR) [20], which is one of the greatest risk factors for increased burden [22]. Yet, the use of supportive services among these caregivers is low [20]. Unfortunately, when family caregivers lack support and resources, they experience greater burden [23]. A multinational review focused on the dementia-related caregiver burden further suggested that the caregiver burden influences time to medical presentation of care recipients, care recipient condition at presentation, and care recipient institutionalization [24]. 

The consequences of being a caregiver of an older adult may be further compounded by the negative influence of dysphagia on the family system. Given an aging population that is forecasted to have an increased number of older adults with complex caregiving needs, this may become even more relevant [25]. The healthcare system will continue to rely on family caregivers, and therefore needs to prioritize supporting them. In light of the far-reaching consequences of the caregiver burden, it is critical that we establish a better understanding of the factors contributing to overall levels of the burden, including a better understanding of the negative effects of dysphagia on caregivers’ daily lives. The purpose of this study was to synthesize the results of published literature on the caregiver burden in caregivers of community-dwelling older adults with dysphagia.

## 2. Materials and Methods

### 2.1. Operational Definitions

This systematic literature review was guided by the methods from the Cochrane group [26]. Operational definitions were determined a-priori; namely, older adult, any adult over the age of 60; community-dwelling, living at home with family; caregiver, an unpaid person caring for and living with an elderly family member; caregiver burden, how a CR’s condition negatively impacts his/her caregiver; and swallowing impairments, all types of swallowing impairments (oral, oropharyngeal, pharyngeal, and esophageal dysphagia) and/or the need for enteral feeds. Eating impairments and situations where the elderly were malnourished and/or had significant oral health needs were also considered, as these are often closely related to dysphagia. Sixty years of age and older was used to define older adults in order to capture as many studies as possible in the dysphagia literature, as many studies, particularly older studies, use this cut-off for the “older adult” age group (e.g., [27,28,29,30]).

During the title and abstract review stage, the definition of swallowing impairments also included primary medical diagnoses known to be associated with swallowing impairments (stroke, dementia, Parkinson’s disease, brain injury, head and neck cancer, amyotrophic lateral sclerosis, multiple sclerosis, chronic obstructive pulmonary disease, and spinal cord injury). This was done in order to capture studies that did not specifically mention swallowing within the abstract, but measured it in the text.

### 2.2. Search Strategy and Inclusion Criteria

A comprehensive literature search was carried out in April 2018 to find reports of burden in caregivers of community-dwelling older adults with dysphagia. The electronic databases that were searched for relevant articles were the following: Medline (1946 onwards), Embase (1974 onwards), Cochrane (2005 onwards), CINAHL (1937 onwards), and Web of Science (1900 onwards). A supplemental search was also conducted in Pubmed. Searches were limited to English-language papers and were conducted by a librarian. Reference lists and citing literature of pertinent articles were cross-checked to ensure all relevant articles were reviewed. The original search strategy (Appendix A) included all years as appropriate per database to 4 April 2018. Search terms were chosen by the authors, who are both speech-language pathologists, and a librarian, and reflect concepts and terminology known to be used in the swallowing caregiver burden, and gerontology research communities.

### 2.3. Study Selection

Only studies with published abstracts were considered for this review. All studies were examined based on the following: (a) they presented original research (i.e., practice guidelines, reviews, position papers, opinion statements, conference proceedings, book chapters, and so on, were not accepted); (b) data from elderly, community-dwelling CRs were easily extracted; (c) caregiver burden was measured; (d) swallowing was discussed; and (e) CRs were not palliative (i.e., receiving end-of-life care) as this brings about unique stressors and burdens for caregivers. Both authors assessed all identified abstracts against the inclusion criteria. Accepted abstracts moved to full article review using the same inclusion criteria, again rated by both authors. Disagreements between raters were resolved by discussion and consensus.

### 2.4. Data Extraction

Data extracted from all accepted articles included the following: type of study, aims, mean age of the CRs and caregivers, and medical diagnoses of the CRs. Data were also collected on tools used to measure caregiver burden and dysphagia, comorbidities, the relationship between dysphagia and burden, and caregivers’ descriptions of experiences relating to dysphagia.

### 2.5. Critical Appraisal

The methodological quality of the accepted studies was assessed using critical appraisal tools from the Joanna Briggs Institute, and chosen based on the type of study (cross-sectional [31], qualitative [32], or cohort [31]), given that the Cochrane systematic review methodology is primarily geared towards randomized-control trials [26].

## 3. Results

### 3.1. Literature Retrieval

**Figure 1:**
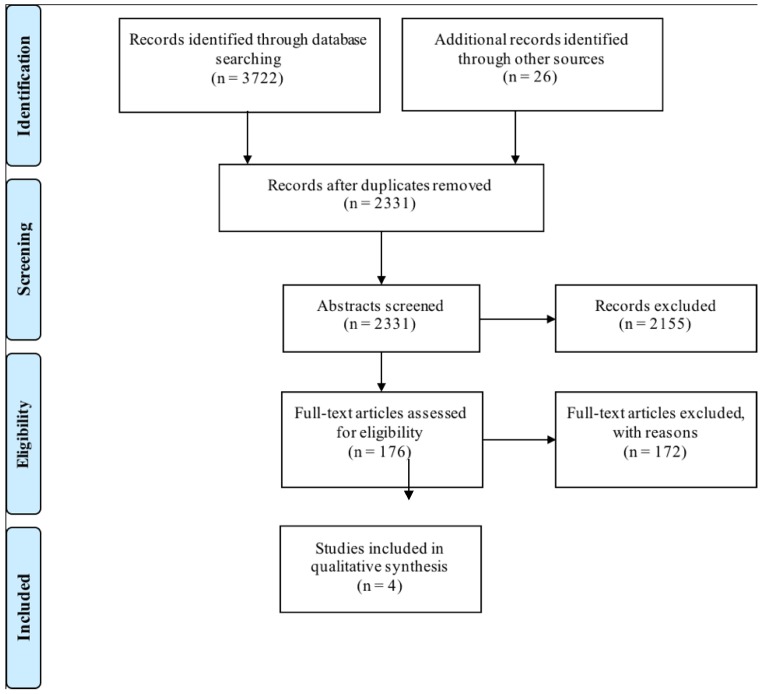
A Preferred Reporting Items for Systematic Reviews and Meta-Analyses (PRISMA) flow diagram depicting the process of retrieving articles for the current systematic review to explicitly indicate the number of records identified, included, and excluded at each stage [33].

The results of the literature search, according to the 2009 PRISMA (Preferred Reporting Items for Systematic Reviews and Meta-Analyses) guidelines for systematic reviews, are summarized in Figure 1 [33]. The initial search yielded 2331 unique publications, which were reviewed to ensure that they were written in English, published in peer-reviewed journal articles, and met the inclusion criteria. Any articles that appeared to focus solely on the institutionalized or hospitalized elderly, and participants under the age of 60, were excluded. Following this step, 176 articles remained for further analysis. Duplicate full-text reviews were completed for all articles using the previously described criteria in order to confirm relevance. Agreement between the two raters before reconciliation on rejecting or accepting abstracts was 93%; agreement on the reasons for rejecting full-texts was 92%.

The article set was narrowed down to a final inclusion list of only four. The reference lists of these articles were screened for any additional titles that suggested that the dysphagia-related caregiver burden may have been discussed, and that may have been missed in the original search. An additional 26 articles were analyzed to determine suitability for inclusion in the final review. Upon detailed review using the same inclusion criteria as previously mentioned, none of the studies contained the necessary data. The majority of articles were excluded from both the original search and the supplemental search because they did not discuss swallowing, or the CRs were not over the age of 60. Some articles had a portion of participants over the age of 60, but these data could not be extracted from the rest of the sample. Other reasons for exclusion included the following: CRs were palliative, caregivers did not live with the CRs, the burden was not measured, and the caregivers were paid. 

### 3.2. Study Characteristics

Study characteristics are depicted in Table 1. Agreement between the two raters was 100% on all data points from 50% of the selected studies, indicating excellent accuracy. Two studies were cross-sectional [34,35], one was qualitative [36], and the fourth was a cohort study [37]. Two of the studies investigated the overall caregiver burden [35,36], while the other two aimed to understand the caregiver burden associated with feeding and swallowing difficulties [34,37]. Three of the four articles studied CRs who had a neurodegenerative disease (Alzheimer’s or Parkinson’s disease) [34,36,37], whereas the fourth study recruited CRs post-stroke [35]. The majority of caregivers were spouses [35,36,37], with only one study recruiting mainly caregivers who were children [34]. In all studies, the mean age of the caregivers was less than that of the CRs.

### 3.3. Methodological Quality

The risk of bias ratings for the cross-sectional [34,35], cohort [37], and qualitative [36] studies are presented in Table 2, Table 3 and Table 4, respectively. An assessment of risk of bias allows one to consider potential biases or flaws in the design of a given study. Potential sources of bias emerged across all studies in the current review. For the cross-sectional studies, the setting where the studies were conducted was unclear and not all outcomes were measured in a valid (measures what it is intended to measure) and reliable (measures are consistent) way. The cohort study failed to identify confounding variables, or outside influences that may have skewed the results, and to describe reasons for why some participants did not complete the entire study (loss to follow-up). The qualitative study lacked clarity in stating the philosophical perspective, or underlying assumptions, in relation to the research methodology, provided no theories which offer an explanation about the main concern of the population and how that concern is resolved, and did not address the influence of the researcher on the study.

### 3.4. Main Findings

The methods for measuring dysphagia differed between the four studies. Two studies concluded that CRs experienced dysphagia based on self-report [35,36], one study used feeding tubes as a proxy [34], and the fourth study used a subsection of the Aversive Feeding Behavior Inventory (AFBI) [38] to determine the presence of dysphagia [37]. While the qualitative study [36] did not report the percentage of participants presenting with dysphagia (dysphagia and choking were a “troublesome symptom and problem” reported by “many” participants), the percentages varied across the remaining studies. Dysphagia was found to be present in 12.9% of stroke survivors [35] and 25.6% of participants with advanced dementia used feeding tubes [34]. The relative frequencies for the behaviors on the AFBI related to swallowing impairments among individuals with Alzheimer’s disease were as follows: fails to open mouth unless physically prompted (3.6% of participants); puckers lips, preventing food entry (4.0%); closes mouth tightly, clenches teeth or lips, preventing food entry (3.6%); continuous tongue or mouth movements, hindering or preventing food entry (13.0%); accepts food then expels it (2.2%); accepts food then fails to swallow (2.2%); accepts food, fails to seal mouth closed, drools food (13.0%); coughs or chokes on food (13.0%); and presents with wet, gurgling voice (14.3%) [37]. Across all of the studies, only presence/absence of dysphagia was reported and no information about severity was presented.

Overall, increased caregiver burden was associated with the presence of dysphagia in CRs across all four studies. Two studies used validated tools to measure caregiver burden: the Zarit scale [37] and the Sense of Competence Questionnaire [35]. The Zarit scale evaluates the level of burden through self-report to respond to 22 questions surrounding personal and role strain [23]. The mean Zarit scale score was 38.9 ± 14.2 (indicative of moderate burden) in the caregivers of CRs who had feeding behaviors that worsened over time, whereas the mean Zarit scale score was 28.8 ± 12.4 (indicative of mild burden) in the caregivers of CRs whose feeding behaviors did not change (*p* < 0.0001) [37]. The Sense of Competence Questionnaire is a questionnaire composed of 35 items intended to measure the perceived caregiver burden [39]. This tool asks questions about the how the personal life of the caregiver is affected, as well how satisfied the caregiver is with their own caregiving and their loved one as a CR [39]. Each item is graded on a four-point scale, with higher scores indicating a higher level of burden. The mean overall Sense of Competence Questionnaire burden score was 2.7 ± 0.5 for caregivers whose CR had swallowing impairments and 2.3 ± 0.6 for caregivers whose CR did not have swallowing impairments (*p* < 0.01) [35].

The other two studies included in this review interviewed caregivers to describe degree of the burden [34,36]. In one article, a caregiver recalled the emotional burden associated with addressing the need for a feeding tube, related both to how the CR would obtain adequate nutrition, as well as the decisions surrounding tube placement [36]. Other participants discussed balancing the risks associated with choking and offering CRs desired foods. The second study that interviewed caregivers specifically asked about the overall burden caregivers felt while caring for their CRs with advanced dementia and the burden of making decisions for their CRs [34]. Over 75% of caregivers whose CRs used a feeding tube reported burden of care to be ‘‘heavy’’ or ‘‘very heavy”. Specifically, 44% of these caregivers reported very heavy burden, compared with 19% of those caring for CRs who did not use feeding tubes (*p* < 0.05, as per the data provided in the original study). Further, there was a trend for caregivers of CRs who used a feeding tube to report a greater burden of making decisions (decisions were “very hard” for 36.7% of these caregivers when compared with 27.0% for caregivers of CRs who did not use a feeding tube).

## 4. Discussion

After a thorough search of the literature and the application of a strict set of inclusion criteria, four studies detailing the caregiver burden experienced by caregivers of community-dwelling older adults presenting with dysphagia were analyzed and synthesized. Overall, the presence of the caregiver burden was associated with dysphagia. Worsening feeding-related behaviors over time were associated with moderate levels of burden, the presence of dysphagia was associated with increased burden. The use of feeding tubes, which can be used as a proxy for severe swallowing difficulties, was more frequently associated with “heavy burden” [34,35,37]. Dysphagia and the necessity for feeding tubes were also associated with increased emotional and psychological burden related to balancing risks associated with choking and allowing CRs to eat desired foods, nutritional intake concerns, and the grieving and acceptance processes related to tube use [36].

While the presence of increased burden was clearly documented, dysphagia-related burden was not the primary focus of the literature analyzed; therefore, the exact nature of the burden and underlying contributors were not delineated. Although not studied specifically among co-residing family caregivers of older adults, previous literature on the influence of dysphagia on caregivers offers suggestions. A number of studies have described outcomes for caregivers of individuals with head and neck cancer, and common themes emerged related to “negotiating a new normal” [12,13,14,15,16]. Caregivers reported increased fear and anxiety related to their responsibilities, their CR’s nutrition status, and choking. Caregivers reported a dramatic shift in daily routines, with nutritional intake increasing schedule rigidity. While feeding tubes eased concerns over nutritional intake, caregivers reported feeling ill-prepared about the new skills and knowledge required [16] and had poorer quality-of-life scores when compared with caregivers of individuals without feeding tubes [15], similar to what was found in an article in the current review [34]. It is critical that we consider how feeding tubes may contribute to the dysphagia-related burden, as they are often prescribed as a result of severe dysphagia. Caregivers in the cancer literature described an increase in conscious thought and intentional activity required for preparing meals. Moreover, meals lacked togetherness as caregivers reported eating alone so as not to upset their CRs. Dysphagia also led to decreased social involvement outside of the home [12,13,14,15,16], and caregivers indicated that they lacked support [13]. Similar findings are reported among caregivers of individuals following stroke and traumatic brain injury [12]. These caregivers were also fearful and uncomfortable trying to balance safety concerns with patient food preferences. Uniquely, these caregivers reported trying to emphasize the positive effects of socializing during mealtimes whenever possible, as they viewed this to be a more important issue than the “actual food”. Caregivers of individuals with neurologically-based dysphagia have also been found to have increased anxiety when compared with caregivers of individuals without dysphagia [40]. Interestingly, a history of previous dysphagia treatment was associated with increased anxiety levels. This may suggest that disease chronicity, despite attempted treatment, plays a role in burden levels.

**Figure 2:**
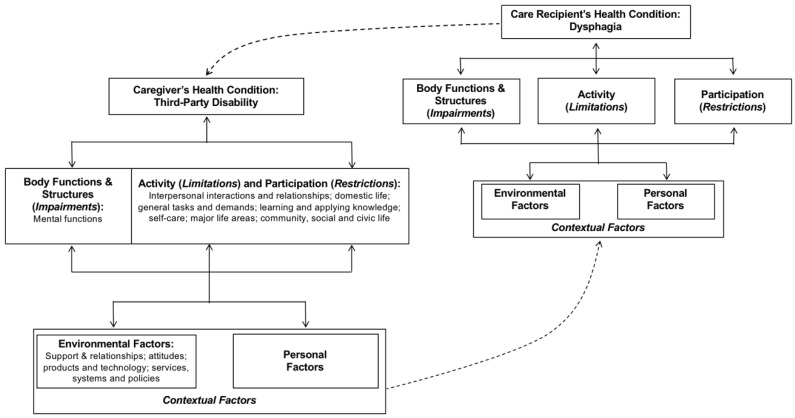
Application of the International Classification of Functioning, Disability, and Health framework [18] to understanding the influence of dysphagia on third-party disability in caregivers, based on the work of Nund and colleagues [14].

Nund and colleagues [14] delineated the third-party disability experienced by caregivers of individuals with dysphagia within the International Classification of Functioning, Disability, and Health (ICF) framework [18] (see Figure 2). Notably, most caregiver comments mapped onto domains and categories related to activities and participation and not just domains pertaining to just eating and drinking (i.e., self-care). Figure 2 also highlights the cyclical influence that a CR’s dysphagia and a caregiver’s third-party disability can have on one another; dysphagia can impact a caregiver’s functioning, activities, and participation in the context of the environment and personal factors, which in turn can act as another contextual factor influencing the health condition (i.e., dysphagia) of the CR.

It is likely that the burden and contributors to the burden experienced by caregivers of older adults with dysphagia mirror those previously reported among other populations. However, older adults also present with some unique challenges that may further complicate the situation. One hallmark of aging is a continual, albeit gradual at times, decline in function. While much of the previous literature among head and neck cancer patients indicates a degree of acceptance related to the “new normal”, the aging process does not allow for such stability to occur. As chronicity may also be associated with increased burden, given this lack of stability, the impact of dysphagia on caregivers of the older adults may be further magnified. Older adults are also often relying on adult children or spouses as caregivers [41,42]. For adult children who are members of the “sandwich generation”, the balance between child rearing, elder care, and work force demands can be challenging [43]. For spouses, they themselves are also aging and present with their own healthcare needs [44]. Yet, the research is lacking. It is unknown whether the influence of dysphagia on caregivers of older adults is indeed similar to what has been previously reported, and whether this varies based on swallowing-related needs (e.g., severity level, tube feedings). It is also unknown whether caregivers of older adults with dysphagia similarly feel a lack of support as related to their new mealtime roles. Additionally, it is not yet clear what role healthcare providers can play in ameliorating the burden related to dysphagia. However, research has shown that there is a high prevalence of dysphagia in older adults in acute care hospitals, and these patients have a higher risk of mortality post-discharge and a higher risk of being discharged to nursing homes [45]. Supporting caregivers may allow these patients to be discharged to home, which in turn may increase their quality of life. Recognizing and addressing the caregiver burden as related to dysphagia is necessary to improve caregiver outcomes and is likely essential for also improving care recipient outcomes.

While it is obvious that the caregiver burden exists among caregivers of community-dwelling older adults who have dysphagia, the exact prevalence and severity of the caregiver burden cannot be determined from the currently available literature. Differing impressions regarding the caregiver burden may be the result of inconsistent use of definitions. Further, measures used to determine the presence of both the caregiver burden and swallowing impairments were varied across the studies included in the current review, and also were quite non-specific. It is evident that studies are needed in which standard definitions of both the caregiver burden and swallowing status are applied in order to determine the presence and severity of both conditions, and the relationship between the two.

### 4.1. Limitations of the Systematic Review

This systematic review looked for peer-reviewed articles in the literature describing caregiver burden associated with swallowing impairments of community-dwelling older adults. However, the articles are a result of a comprehensive search strategy using specific keywords and medical subject (MeSH) headings (Appendix A). Our results may have been further limited by our inclusion criteria, as we only included studies that reported caregiver burden and swallowing impairment data in a way that allowed for data extraction. A large majority of articles found in our initial search did not have the information required for inclusion. Lastly, only 50% of articles were reviewed in duplicate during our data extraction process. There was perfect agreement for this process across raters; however, decisions made by one rater may have excluded qualitative data that a second rater may have judged to be relevant. 

### 4.2. Conclusions

Our careful review of the literature regarding the caregiver burden among caregivers of community-dwelling older adults presenting with dysphagia across four studies has provided a descriptive profile of the current evidence of the caregiver burden in this population. Because of the extreme divergence of methods used to measure both the caregiver burden and swallowing impairments across the included studies, it was not possible to reach objective conclusions about the prevalence or common causes of the caregiver burden. Previous research in both head and neck cancer and neurologic populations has pointed to several potential causes of burden, all of which may be applicable to caregivers of community-dwelling older adults; however, further research is required to confirm this.

The current study brings to light the paucity of evidence describing the impact of dysphagia on the caregiver burden and argues for the need of further investigations that carefully apply accepted diagnostic criteria. Future research should focus on the standardization of both terminology and measurement instruments, given that this review revealed that several operational definitions and tools are being used to diagnose dysphagia and the caregiver burden. The use of valid and reliable tools to measure both the severity of the caregiver burden and swallowing function is necessary to understand how swallowing impairments contribute to the caregiver burden and to develop evidence-based interventions to support caregivers, and in turn reduce their level of burden when caring for community-dwelling older adults. Ultimately, dysphagia is a chronic health condition that occurs in context requiring a more comprehensive approach to management. Successful intervention to limit the caregiver burden has the potential to therefore improve the quality of life for both caregivers and care recipients, and possibly allow community-dwelling older adults to reside at home for longer before entering care facilities.

## Figures and Tables

**Table 1:** Characteristics of all studies meeting criteria for inclusion in current systematic review.

Study	Design	Study Aim(s)	N (CG/CR Dyads)	Mean Age of CRs (years) ^a^	Mean Age of CGs (Years) ^a^	Diagnosis of CRs	Relationship of CGs to CRs
Bentur, N., Sternberg, S., Shuldiner, J., & Dwolatzky, T. (2015) [34]	Cross-sectional	To examine the prevalence of feeding tube use among OPAD living in the community in Israel. To describe the demographic, social, and medical characteristics of OPAD and to compare feeding tube users and nonusers. To describe the quality of care of OPAD and the burden of care on their caregivers, and to compare feeding tube users and nonusers.	117	86.5 (7.7)	61.7 (12.5)	Advanced dementia	74% children, 22% spouses
Choi-Kwon, S., Kim, H-S, Kwon, S. U., & Kim, J. S. (2005) [35]	Cross-sectional	To identify the factors related to caregiver burden in chronic stroke survivors in Seoul, Korea.	154	61.9 (8.1) ^b^	54.5 (13.1)	Chronic ischemic stroke	70% spouses, 14% children, 14% daughters-in-law
Habermann, B., & Shin, J. Y. (2017) [36]	Qualitative	To explore the needs, concerns, and preferences of couples with advanced PD in the United States of America as they plan the care needed for the future.	14	73.31 (9.3)	72.13 (8.8)	Advanced Parkinson’s disease	100% partner or spouse
Riviere, S., Gillette-Guyonette, S., Andrieu, S., Nourhashemi, F., Lauque, S., Cantet, C., Salva, A., Frisoni, G., & Vellas, B. (2002) [37]	Cohort	To investigate predictors of aversive feeding behaviors which occurred during a one-year interval among AD patients living at home with a caregiver in France, Spain and Italy.	193	76 (8.1)	61.1 (13.4) and 62.5 (12.5)—study divided CGs into 2 groups	Alzheimer’s disease	54% spouses, 35% another relative

Note: ^a^ The majority of the studies reported ages only as mean years with standard deviation. Thus, age ranges and medians are not presented here. ^b^ No significant difference was found in overall caregiver burden for caregivers of individuals over the age of 62 years and under the age of 62. Thus, this sample does include care recipients under the age of 60. AD = Alzheimer’s disease; CG = caregiver; CR = care recipient; N = total number; OPAD = older people with advanced dementia; PD = Parkinson’s disease. Ages are presented as mean years with standard deviations in parentheses.

**Table 2:** Joanna Briggs Institute risk of bias ratings for cross-sectional studies [31].

Joanna Briggs Guidelines	Bentur, N., Sternberg, S., Shuldiner, J., & Dwolatzky, T. (2015) [34]	Choi-Kwon, S., Kim, H-S, Kwon, S. U., & Kim, J. S. (2005) [35]
Were the criteria for inclusion in the sample clearly defined?	Yes	Yes
Were the study subjects and the setting described in detail?	Subjects—yes; setting—no	Subjects—yes; setting—no
Was the exposure measured in a valid and reliable way?	N/A	N/A
Were objective, standard criteria used for measurement of the condition?	Yes	Unclear
Were confounding factors identified?	Yes	Yes
Were strategies to deal with confounding factors stated?	Yes	Yes
Were the outcomes measured in a valid and reliable way?	No	No
Was appropriate statistical analysis used?	Yes	Yes

**Table 3:** Joanna Briggs Institute risk of bias ratings for the cohort study [31].

Joanna Briggs Guidelines	Riviere, S., Gillette-Guyonette, S., Andrieu, S., Nourhashemi, F., Lauque, S., Cantet, C., Salva, A., Frisoni, G., & Vellas, B. (2002) [37]
Were the two groups similar and recruited from the same population?	Yes
Were the exposures measured similarly to assign people to both exposed and unexposed groups?	N/A
Was the exposure measured in a valid and reliable way?	Yes
Were confounding factors identified?	No
Were strategies to deal with confounding factors stated?	No
Were the groups/participants free of the outcome at the start of the study (or at the moment of exposure)?	N/A
Were the outcomes measured in a valid and reliable way?	Yes

**Table 4:** Joanna Briggs Institute risk of bias ratings for the qualitative study [32].

Joanna Briggs Guidelines	Habermann, B., & Shin, J. Y. (2017) [36]
Is there congruity between the stated philosophical perspective and the research methodology?	Unclear
Is there congruity between the research methodology and the research question or objectives?	Yes
Is there congruity between the research methodology and the methods used to collect data?	Yes
Is there congruity between the research methodology and the representation and analysis of data?	Yes
Is there congruity between the research methodology and the interpretation of results?	Yes
Is there a statement locating the researcher culturally or theoretically?	No
Is the influence of the researcher on the research, and vice-versa, addressed?	No

## Appendix A

**Table A1:** Medline search strategy. The asterisks (*) were used to retrieve variations on a distinctive word stem or root.

Search Number	Search Terms
1	Caregivers/
2	(caregiving or “care giving” or caregiver* or care giver* or carer or carers).tw,kw.
3	1 or 2
4	exp aged/or “aged, 80 and over”/or frail elderly/
5	Geriatrics/
6	(geriatri* or gerontol* or elder? or elderly or senior* or senescen* or septuagenarian* or octogenarian* or nonagenarian*).tw,kw.
7	((old* or aged*) adj2 (adult* or people or patient* or person* or women or men or individual*)).tw,kw.
8	or/4-7
9	3 and 8
10	exp Deglutition Disorders/
11	Deglutition/
12	dysphagi*.tw,kw.
13	deglut*.tw,kw
14	swallow*.tw,kw.
15	or/10–14
16	Nutrition disorders/
17	exp Malnutrition/
18	Nutritional physiological phenomena/
19	Nutritional Status/
20	exp Nutrition Therapy/
21	Feeding Behavior/
22	exp Diet/
23	exp Eating/
24	exp Food Habits/
25	nutrition*.tw,kw.
26	malnutrition*.tw,kw.
27	malnourish*.tw,kw.
28	(meal* or dinning or dinner*1 or supper*1 or lunch*2 or breakfast*1).tw,kw.
29	(eat or eating).tw,kw.
30	(feed or feeding).tw,kw.
31	(drink*1 or drinking).tw,kw.
32	or/16–31
33	Feeding Methods/
34	Enteral Nutrition/
35	exp Parenteral Nutrition/
36	((enteral or parenteral) adj2 (feed* or nutrition*)).tw,kw.
37	((force or tube or intravenous) adj2 feed*).tw,kw.
38	or/33–37
39	9 and 15
40	9 and 32
41	9 and 38
42	Elder Nutritional Physiological Phenomena/
43	3 and 42
44	39 or 40 or 41 or 43
45	44 not (exp animals/not exp humans/)
46	limit 45 to English language
